# Exploring the correlation between rising temperature and household electricity consumption: An empirical analysis in China

**DOI:** 10.1016/j.heliyon.2024.e30130

**Published:** 2024-05-03

**Authors:** Yong Su, Kaleem Ullah

**Affiliations:** a School of History, Xinjiang University, Urumqi, Xinjiang, 830046, China; b National University of Sciences & Technology, Islamabad, Pakistan

**Keywords:** Rising temperature, Household electricity consumption, China, Residential energy consumption

## Abstract

This study investigates the nexus between rising temperatures and household energy consumption using data from respondents' electricity bills in the "China Residential Energy Consumption Survey." Our analysis reveals a significant correlation, with an 8.9 % increase in yearly energy consumption observed when the average temperature exceeds 32 °C. Additionally, we explore potential shifts in power usage due to global warming by integrating baseline estimates with daily temperature forecasts from eight contemporary climate models. Our findings project alarming trends: without interventions to curb greenhouse gas emissions, home electricity consumption could surge by 9.59–30.09 % in the medium term and by 9.77–47.70 % in the long run. By shedding light on these critical connections, our research underscores the urgent need for policy actions to mitigate the adverse impacts of climate change on energy consumption patterns.

## 1 Introduction

The interplay between climate change and energy consumption has gained significant importance today because of the heightened environmental issues. Given the escalating global temperatures, comprehending this phenomenon is paramount in efficient energy strategizing and mitigating climate change [1]. China, the foremost energy consumer and the largest emitter of greenhouse gases globally, assumes a substantial responsibility [2]. Urbanization and substantial expansion of the middle class within the nation resulted in heightened energy requirements, and there has been a notable lack of comprehensive and empirical investigations about consumption patterns in connection with climate change [3].

The increasing effects of climate change have sometimes resulted in reduced regular weather patterns. The present viewpoint among many researchers challenges the claim made by the Intergovernmental Panel on Climate Change (IPCC) that, even with the stabilization of greenhouse gas emissions, global temperatures will not continue to rise indefinitely [4]. Simultaneously, extreme temperature increases motivate individuals to seek shelter indoors. Nevertheless, considering the current global energy resource distribution, the precise consequences of severe weather events on household electricity usage remain uncertain.

The impact of thermal escalation on domestic electricity consumption in China. Our study focuses on China, the most significant source of greenhouse gases worldwide and highly vulnerable to climate change consequences. Understanding the effects of elevated temperatures on residential electricity usage is of utmost importance for various reasons. The initial rise in carbon dioxide (CO2) emissions, a significant factor in China's local air pollution and global climate change, may be further exacerbated by the heightened energy demand resulting from elevated temperatures. Given that oil accounts for over 70 % of China's electricity generation, it is reasonable to hypothesize that this fossil fuel plays a substantial role in the country's total carbon dioxide emissions [5]. An increase in residential power usage due to extreme heat is expected to contribute to higher levels of CO2 emissions, which will worsen the effects of climate change unless changes are made to the energy combination. The relationship between heat increases and household power consumption has significant societal ramifications. Low-income households are especially vulnerable to energy poverty due to their need to adapt to extreme temperatures and the increased costs associated with cooling and heating. Power supply management is crucial in maintaining optimal performance during temperature extremes. Including such information is crucial for making informed decisions regarding investments in peak generation capacity, which can effectively address potential power shortages in the future.

In the China Residential Energy Consumption Survey (CRECS), which includes verified monthly power usage for 8534 households in China from 2018 to 2021, our analysis revealed an asymmetrical relationship between perpetual temperatures and energy consumption. This finding is consistent with previous study conducted by Ref. [6]. The power consumption experiences a significant escalation on days when the average temperature surpasses 32 °C. This results in an annual rise of 8.9 % in electricity consumption for each additional day with a mean temperature above 32 °C, compared to days ranging from 10 °C to 16 °C. On the other hand, it is worth noting that the rise in power consumption exhibits a relatively diminished magnitude when temperatures surpass 12 °C in contrast to colder periods.

The existing body of literature examining the correlation between rising temperatures and household electricity consumption provides valuable insights into the intricate dynamics influencing energy usage patterns within the context of climate change [7]. underscore China's pivotal role as the world's largest emitter of greenhouse gases and its susceptibility to the consequences of climate change. The rapid urbanization and the expanding middle class in China have significantly contributed to heightened energy demands, emphasizing the critical need to comprehend the ramifications of elevated temperatures on residential electricity usage [8].

Recent studies have spotlighted the substantial impact of air conditioning units (ACs) on the surge in household electricity consumption during periods of elevated temperatures [9]. The inclination of households to acquire ACs, often opting for multiple-unit ownership, accentuates the necessity for empirical investigations to discern the specific impact of heatwaves on power consumption [10]. While prior research has endeavored to evaluate temperature impacts using metrics such as cooling and heating degree days, limitations arising from region-specific definitions and arbitrary baseline temperatures have spurred the exploration of alternative methodologies [11]. Adopting a semi-parametric approach in current studies facilitates a more nuanced comprehension of the relationship between temperature ranges and electricity usage, effectively addressing gaps identified in earlier research efforts.

Our research unveils a pivotal transmission mechanism through which elevated temperatures significantly contribute to heightened power consumption, primarily attributed to the increased usage of air conditioning units (ACs) [12]. In response to rising temperatures, households exhibit a discernible inclination to acquire ACs, often opting for multiple units. This study adds a novel dimension to existing knowledge by meticulously examining the intricate relationship between heat waves and elevated power consumption. Our findings reveal that, in the medium term, domestic energy consumption can surge by 8.87–29.16 %, and in the long run, this increase could reach 8.88–46.65 %. Notably, these projections are contingent upon implementing policy interventions geared toward reducing greenhouse gas emissions.

Our study contributes to the literature by dissecting responses to global warming at the level of individual appliances within China, offering insights into household-level energy consumption patterns. By incorporating the latest climate change models and future temperature forecasts, our analysis extends beyond mere forecasting to provide actionable insights for policymakers and stakeholders aiming to mitigate the impacts of rising temperatures on electricity consumption.

Our methodology, characterized by its nonlinearity and non-parametric nature, is distinctive. This approach enables an effective modeling and understanding of the impacts of extreme temperature events on power consumption. While previous research has endeavored to assess temperature impact using cooling and heating degree days, the limitations imposed by region-specific definitions and arbitrary baseline temperatures prompted us to employ a semi-parametric methodology. This allows for a nuanced assessment of the impact of different temperature ranges on electricity usage, addressing constraints prevalent in prior studies.

Remarkably, our study achieves a pioneering distinction by dissecting responses to global warming at the level of individual appliances within a vast and geographically diverse country like China [10,13,14]. In contrast to prior research relying on household variables such as affluence, residence features, or equipment stocks to forecast domestic power consumption without accounting for actual usage or energy efficiency, our approach delves into the specificities of appliance-level responses. Furthermore, while other studies have assessed the role of cooling systems in mitigating the effects of global warming, our study surpasses them by providing detailed insights into the use and efficacy of freezing devices at the household level.

In a forward-looking dimension, I incorporate the latest future temperature forecasts into our calculations, replicating power usage increases based on the most recent climate change models. This forward-thinking approach enhances the robustness and relevance of our findings, positioning our research at the forefront of unraveling the complex interplay between temperature dynamics and household electricity consumption.

The rest of the paper is structured as follows: Section 2 presents the dataset and empirical approach of the paper; Section 3 presents the results and analysis, simulation of future electricity, impact of extreme temperatures, and robustness check of the paper; Section 4 presents the conclusion and policy implications.

## 2 Dataset and empirical approach

### 2.1 CRECS

The Renmin University of China has undertaken the "China Residential Energy Consumption Survey (CRECS)" since 2012, offering valuable insights into residential electricity usage and appliance types. The comprehensive survey spanned 314 cities and 26 provinces in mainland China, with the sample evenly distributed among provinces based on the 2010 Sixth National Population Census. A distinguishing feature of CRECS is its requirement for power bills or other evidence of power usage, reducing measurement error. Additionally, the survey mandates that all members of an eligible household be present for at least six months annually, ensuring a realistic portrayal of typical use patterns. This approach contrasts with the US Residential Energy Consumption Survey.

The CRECS encompasses data on electricity prices, metering, billing, household demographics, and housing features. Our study leverages CRECS surveys from 2018 to 2021, amalgamating them to create a repeated cross-sectional sample comprising 8534 homes post-cleaning. To enhance the transparency of our data section, we provide clear explanations for variable selection, emphasizing their relevance. Unlike previous studies, CRECS includes information on power bills and mandates household members' presence for six months yearly, contributing to a more accurate representation of usage patterns.

Panel A of Table 1 presents summary statistics on energy use and prominent home equipment for the sampled families. On average, households in the sample consumed 1332.2 kWh of power annually at 411.18 RMB, constituting 2.9–3.23 percent of total expenditures per person. Despite variations in annual power usage and geographic location, households in China's warmer and humid southern regions exhibited higher power consumption.

**Table 1:** Quantitative description.

	Mean	Std. Deviation	Min	Max	Mean Urban	Mean Rural	Mean southern	Mean Northern
**Panel A**								
Annual Electric Use (in kilowatt-hours)	1332.2	924.464	1	4800	142.287	1227.79	1411.188	12.876
Electric Use during the Summer (kilowatt-hours/month)	2212.2	172.237	1	800	222.267	166.656	232.206	204.406
Monthly Winters Electricity Use (kWh)	169.43	123.355	1	400	214.4	166.657	14.442	162.219
Cost of Electric (in Yuan)	411.18	404.393	1	1996.7	411.101	402.247	423.361	394.43
Cost of electric in Yuan per kilowatt-hour	1.261	1.33	2	2.25	1.241	1.184	1.163	1.16
Pricing of electricity by province in RMB per kilowatt-hour	1.255	1.32	1.175	1.489	1.234	1.279	1.254	1.256
Ownership rate of ACs	1.278	1.385	1	2	1.228	1.377	1.219	1.232
No. of Acs	1.464	1.591	1	4	1.754	0.782	0.265	0.286
Utilization rate of ACs (month/year) b	3.456	2.25	2	1	1.386	1.317	1.907	1.541
Daily usage of ACs (hour) b	5.517	3.375	2	8	6.743	2.284	1.152	1.198
No. of water heaters	1.219	3.436	1	2	1.264	1.221	1.671	1.259
No. of refrigerators	1.445	1.372	1	2	1.802	1.706	1.75	1.239
Quantity of Washers	1.265	1.278	1	2	1.924	1.226	1.223	3.613
TVs in Number	2.377	1.361	1	3	2.225	2.245	2.215	3.335
Quantity of PCs	2.352	1.566	1	4	2.745	1.222	2.221	1.287
The mean efficiency score of ACs c	1.101	1.783	2	4	3.498	3.304	1.161	4.418
The mean efficiency score of other home appliances s	3.212	2.342	2	4	5.6	3.22	1.225	3.21
**Panel B**								
number of days with temperatures above 11°	5.469	22.209	1	113	6.539	5.428	1	15.417
Day Count (−11°, −6)	3.25	2.268	1	56	6.646	4.476	1	12.261
Period [28° 31°]	37.878	32.528	1	143	38.808	32.2286	51.144	14.464
Days [at 31° above]	2.424	2.358	1	23	2.262	2.367	1.865	1.316
**Panel C**								
Family breadwinner sex (Men = 2)	1.285	1.265	1	2	1.233	1.82	1.583	1.4888
Number of years a household head has spent in school.	5.419	2.228	1	23	5.419	3.329	4.373	9.919
Principal breadwinner's age (in years)	52.358	13.386	4	102	52.285	53.316	57.772	54.378
Number of people living in the household.	4.563	2.477	1	15	4.457	1.136	4.499	5.523
Types of Income Received by Households	7.871	1.167	2	16	7.822	3.418	2.209	5.531
House Area (m2)	56.651	82.718	5	1040	56.584	64.472	64.411	53.395
Heaters for the public (Yes = 2)	2.373	1.278	1	2	1.189	1.183	1.118	1.249
The sum of people older than 61.	1.236	3.329	1	3	1.237	1.235	1.282	1.276
Count of people younger than 13 years old.	1.216	1.252	1	4	1.196	1.23	1.214	1.219

The correlation between the usage of air conditioning devices and the quantity of electricity consumed to cool a specific space is highly interrelated. In our study, only a meager 35 % of the homes had air conditioning units installed. Nonetheless, the normative total of cooling systems per domicile was 0.564, implying that specific residences possessed more than one air conditioning apparatus. As a result of the warmer climate, many homes in the South have air conditioners and use them more frequently. The high initial and ongoing air conditioning costs have resulted in about half as many rural homes having air conditioning as urban homes. Rural and urban areas exhibit a significant discrepancy in the ownership of water heaters, another method of dealing with heat stress [15]. Ownership of other vital household appliances, such as freezers, televisions, and "washing machines", displays much narrower disparities. Urban and rural families use these other devices in similar ways. A cursory look at the figures implies that air conditioning ownership and usage will likely contribute to higher power consumption among southern and urban families.

### 2.2 Everyday weather information

The CMA is the source of information on China's weather, and we collect data from a network of 820 synoptic weather stations that record a range of parameters. We use this data, collected continuously between 2018 and 2021, to minimize bias in spatial interpolation of meteorological parameters. The meteorological information from synoptic stations undergo stringent quality standards, with an estimated accuracy of approximately 100 % and a missing data rate of less than 0.1 %. Our spatial interpolation method employs inverse distance weighting to extrapolate meteorological data. Closer stations are given more weight, which results in more accurate outcomes. We ensure adaptability by dividing the yearly daily mean temperatures into ten groups. On average, extreme heat occurs 1.524 days across all counties, with approximately three days of extreme heat every year in southern China.

Interestingly, heat and cold extremes appear more common in rural homes, indicating environmental stress. The county-level meteorological variables are condensed in Panel B of Table 1. We also place drop stations at substantially higher altitudes than typical residential locations to improve accuracy. Although synoptic stations are located throughout China, there is no particular pattern, and many CRECS counties lack a single weather station.

The comprehensive specifications consist of a regulation that oversees the housing and family attributes, consolidated in the ultimate section of Table 1. The average Chinese household comprises three individuals and utilizes approximately 60 square meters of floor space. The majority of the primary earners are men who are in their early fifties and possess a lesser degree of formal education. Public heating is only accessible to more than 17 percent of the population in the northern regions of China. Due to China's one-child policy and swiftly aging populace, senior citizens represent a more considerable percentage of households than youngsters below twelve.

### 2.3 Predictions of climate change

The most recent evaluation report from the IPCC did not depict future climate forecasts using the "Coupled Model Intercomparison Project (CMIP6)". The six GCMs predicted the monthly minimum and maximum temperature values for four "Shared Socio-economic Pathways (SSP) – SSP126, SSP245, SSP370, and SSP585.8." It appears that these values are a precise representation of the medium and long-term timeframes. The temperature data are obtainable for all Chinese regions, and we utilized forecasts from eight GCMs with a spatial resolution of 2.5 min. Focusing on the medium and long-term future, we exclude "SSP126 and SSP585,” representing two severe emission trajectories. SSP126 envisions an extra equitable and sustainable growth pathway with minimal climate change extenuation and adaptation obstacles.

In contrast, the "business as usual" scenario adopted by SSP585 would permit greenhouse gas emissions to continue rising unchecked throughout this century. We employ the methodology [16,17] established to estimate future daily mean temperatures at the county level in three phases. First, we generate monthly mean temperatures and probability distribution functions for all counties using daily mean temperatures from 2018 to 2021. Second, we analyze the anticipated monthly averages and compare them to the average historical monthly means for each region to identify monthly average temperature fluctuations. Finally, for SSP126 and SSP585, we assume that the projected daily mean temperature distribution is similar to its historical distribution and build the projected daily mean temperature distribution for each county over medium and long durations.

### 2.4 Empirical approach

The following specifications are estimated to investigate the impact of daily temperatures on yearly power use as in Eq. (1):

(1) $$\ln\_Elecons_{ijt}=\sum_{m}\;\beta^{m}Temp_{jt}^{m}+\delta^{\prime }Z_{it}+\varphi^{\prime }S_{ijt}+\gamma^{\prime }F_{jpt}+\varepsilon_{ijt}$$

**Fig. 1:**
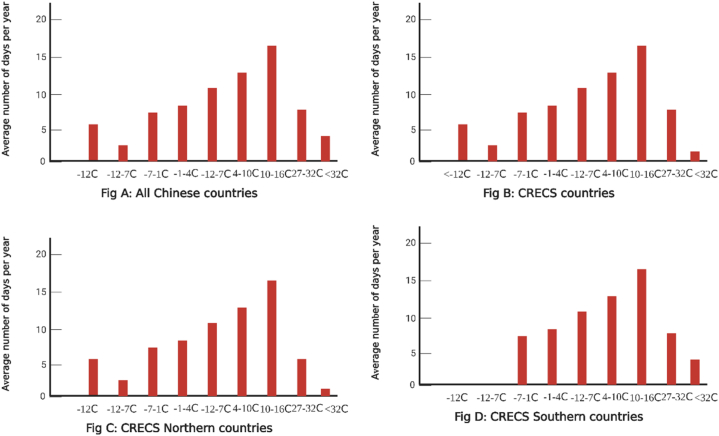
The 2018–2021 Temperature Distribution daily.

We employ a method of partitioning the plausible range of daily mean temperatures. $\sum_{m}\;Temp_{jt}^{m}$ Into m distinct partitions. This remarkable semi-parametric technique provides versatile estimates of non-linear effects across a broad spectrum of daily temperature values while limiting the functional form of the underlying variables. We expose the annual distribution of daily temperatures in degrees Celsius, partitioning them into ten groups: (below 12 °C), (7 °C–1 °C), (4 °C 10 °C), (16 °C 21 °C), (27 °C 32 °C), and (32 °C above). To illustrate, on a given year t and in a county j, the quantity of days with a daily mean temperature below 12 °C is referred to as Temp_jt_. Fig. 1 displays the average daily temperature distributions for the four samples over three years. Notably, all Chinese counties are encompassed within the same distribution as those scrutinized by CRECS (see Fig. 1 panel B), hinting that the counties analyzed by CRECS are emblematic of the climatic diversity throughout China (see Fig. 1 panel A). Based on our statistical summaries, the number of days with mean temperatures below one °C is significantly higher in northern Chinese areas (see Fig. 1 panel C) than in southern ones (see Fig. 1 panel D).

To avoid issues with multicollinearity, we have omitted the reference temperature bin [10 °C 16 °C]. Our semi-elasticity βm, which indicates the marginal impact of an additional day with the temperature in bin βm compared to a day in the reference temperature bin, has been determined through a comparison of homes within the same county, as household fixed effects cannot be accounted for in our repeated cross-sectional data. Despite assuming temperature as an exogenous variable, other factors may still impact our estimations, such as families relocating to areas with lower cooling and heating needs when struggling with high temperatures. To settle this problem, we have incorporated a vast range of characteristics that are acknowledged to impact migration choices, such as family volume and composition, as well as the age and education level of the head of the household. We have utilized "temperature shocks," short-run departures from the longer-run climate pattern, to enhance the causal identification of temperature effects, and [18] bounding technique to ensure the resilience of our preferred specifications to omit parameter bias.

Our Z_it_ "control vector" encompasses a range of variables, including atmospheric pressure and annual precipitation levels at the county level. Meanwhile, our $S_{ijt}$ Variables focus on individual homes, rooms, and appliances. We use a set of fixed effects known as $F_{jpt}$, to account for county, year, or province-specific changes over time. County-fixed effects look at a county's stable characteristics, such as topography, while year-fixed effects capture national-level shocks. To better adjust for province-specific yearly shocks, we utilize year-by-province fixed effects. As the error term εijt may have spatial correlation, we "cluster standard errors" at the county level. Our analysis also explores alternative municipal, municipal-by-year, and provincial clustering methods to ensure robustness.

## 3 Results and analysis

### 3.1 Main outcomes

Table 2 presents estimations derived from Eq. (1), with the temperature mean ranging from 10 to 16 °C as the reference category for all computations. Initially, we restricted the analysis to county and year-fixed effects, eliminating other factors. Column (2) introduces regional diversity in yearly shocks by replacing year-by-province fixed effects with year-fixed effects. Subsequent columns (3) to (6) gradually incorporate additional climate controls, demographic data, energy efficiency of essential household equipment, and house features.

**Table 2:** Baseline outcomes.

(below −10°)	1	2	3	4	5	6
	−1.104	1.11	−1.101	1.106	−1.116	1.1
	−1.117	−1.113	−1.127	−1.126	−1.125	−1.13
[−10 °C–5 °C)	1.106	−1.104	1.106	1.108	1.121	1.12
	−1.122	−1.121	−1.119	−1.119	−1.118	−1.12
[−5 °C–1 °C)	1.107	1.105	1.115	1.114	−1.103	−1.114
	−1.114	−1.115	−1.11	−1.11	−1.11	−1.12
[−1 °C 4 °C)	1.117	1.113	1.109	1.113	1.102	1.112
	−1.112	−1.115	−1.119	−1.118	−1.111	−1.12
[4 °C 10 °C)	−1.101	1.106	1.105	1.105	−1.110	−1.107
	−1.105	−1.106	−1.115	−1.113	−1.109	−1.11
[10 °C 21 °C)	1.141⁎⁎⁎	1.150⁎⁎⁎	1.150⁎⁎⁎	1.147⁎⁎⁎	1.122⁎	1.113
	−1.111	−1.112	−1.118	−1.116	−1.111	−1.12
[21 °C 27 °C)	1.170⁎⁎⁎	1.176⁎⁎⁎	1.167⁎⁎⁎	1.170⁎⁎⁎	1.152⁎⁎⁎	1.164⁎⁎⁎
	−1.121	−1.121	−1.124	−1.122	−1.111	−1.12
[27 °C 32 °C)	1.179⁎⁎⁎	1.193⁎⁎⁎	1.184⁎⁎⁎	1.186⁎⁎⁎	1.171⁎⁎⁎	1.182⁎⁎⁎
	−1.127	−1.123	−1.12	−1.119	−1.113	−1.12
[32 °C above)	1.181⁎⁎⁎	1.197⁎⁎⁎	1.191⁎⁎⁎	1.191⁎⁎⁎	1.188⁎⁎⁎	1.189⁎⁎⁎
	−1.124	−1.13	−1.122	−1.12	−1.13	−1.13
No of Observation	7316	7316	7316	7196	6817	6127
Adj-*R2*	0.316	0.306	0.310	0.319	0.362	0.367
Country FE	Yes	Yes	Yes	Yes	Yes	Yes
Year FE	Yes	No	No	No	No	No
FE Data, by Province, Year	No	Yes	Yes	Yes	Yes	Yes
Climate Regulations	No	No	Yes	Yes	Yes	Yes
Label for Energy Effectiveness	No	No	No	Yes	Yes	Yes
Population Regulation	No	No	No	No	Yes	Yes
Home Automation Systems	No	No	No	No	No	Yes

Notes: The dependent variable is the yearly power usage, which is represented logarithmically. The reference temperature bin of [10–16 °C] has been excluded to prevent "multicollinearity". The weather is affected by various factors, like the average yearly atmospheric pressure, humidity, wind speed, exposure to sunlight, and total yearly rainfall. To ensure energy efficiency in household equipment, the Energy Star label adjusts the standard. If an appliance's energy efficiency rating is unavailable, the median figure across all counties in the United States is used. The variables considered while making adjustments include the gender and educational background of the head of the household, the number of individuals residing in the household, the number of individuals above the age of 60, and the number of children under 12 years old. The age and size of the home also play a role in the adjustments. County-wise, the standard deviations tend to cluster.

Our observations indicate that warm days correlate with a notable increase in power consumption while freezing days have a negligible influence. This finding contrasts with earlier studies by Refs. [19,20], which proposed a U-shaped correlation between temperature and power usage. However, our research reveals a different pattern.

During winter, individuals residing in northern regions experiencing the coldest temperatures benefit from free public heating powered by coal. Notably, our sample highlights that most rural families rely on conventional fuels such as fuel and straw [21], while approximately 35 % of urban households in the north depend on coal-fired public heating.

Based on the specifications we have selected in column (6), the consumption of power surges considerably once the daily average temperature exceeds 21 °C, and this trend is more pronounced in the higher temperature brackets. Our assessments are more substantial than those of previous studies that used the same modeling technique [22]. found that above 32°, home power usage in Shanghai rose by 5.7 % daily using a sample of homes. Monthly power consumption among Mexican homes increased by 3.2 % due to the same transition, according to research by Refs. [[23], [24], [25]].

[26] examined the effect of severe temperatures on energy usage, which is significantly lower and temporary. The investigation of data from all states in the US revealed that energy consumption showed an annual rise between 0.2 % and 0.4 % on days when temperatures reached over 32 °F. Our calculations provide some context, with each family in our sample using an average of 1332.2 kWh of power last year. An additional day of temperatures over 32 °C has the same impact on yearly power usage as an additional day of temperatures between 10 and 16 °C. This slight increase indicates the impact of only one day of extreme heat on a typical family, but the cumulative effect would be substantial. If each family experienced an additional day with temperatures over 32 °C in 2018, domestic power usage in China would increase by 6.3 %, or 44.63 TWh. Our somewhat significant estimates suggest that people adapt to hot days. As per the findings of [27], the correlation between temperature and financial outcomes over a long period can be modeled using correlations derived from a repeatedly "cross-sectional dataset", as done in this research. Another potential explanation is that families permanently increase their power usage by installing air conditioners during hot days. Due to the convenience of already installed air conditioners, people may begin using them even on moderately warm days, causing a significant increase in power consumption.

### 3.2 Heterogeneity

Table 3 provides a detailed breakdown of the diverse aspects, with the first and second columns featuring estimates for northern and southern China, respectively. In keeping with the conclusions reached [28,29], it seems that households in the South tend to experience a U-shaped relationship between their annual average power usage and temperature due to their heavy reliance on electric heaters for warmth [[30], [31], [32]]. As per our examination, power utilization increments by 9.4 percent for every extra day, with average temperatures going from 1 to 4 °C. The temperature in January typically freezes along the line between the Huai River and the Qinling Mountains, which makes public heating a top priority in this region. The conducted simulations by Refs. [33,34] exhibit the likely gains of public heating. These gains encompass a 32 % reduction in energy consumption for southern homes and an improvement in interior comfort during the winter. Providing access to public heating would, therefore, enhance southern residents' well-being.

**Table 3:** Heterogeneity outcomes.

(below −10°)	1	2	3	4	5	6	7	8
	Southern	Northern	Urban	Country	High−income	Low−income	Household Kids and Aging	Households without kidsand Aging
	1	−2.388	2.277	1.12⁎⁎⁎	1.14	1.123	1.101	−1.148
**[−10 °C -5 °C)**	(.)	−2.365	−2.228	−1.122	−1.13	−1.12	−1.114	−1.141
	1	1.161	2.255	1.142⁎⁎⁎	1.192⁎⁎	1.116	−1.104	1.128
**[−5 °C -1 °C)**	(.)	−1.199	−2.266	−1.11	−1.14	−1.121	−11.113	−1.122
	−1.299	−1.329⁎	−2.213	1.147⁎⁎⁎	−1.133	1.122	2.212⁎	−1.103
**[−1 °C 4 °C)**	−1.244	−1.175	−2.231	−1.105	−1.136	−1.116	−2.207	−1.123
	1.194⁎⁎⁎	1.209	2.266⁎⁎⁎	1.11	1.191⁎⁎⁎	1.109	−2.217⁎⁎	−1.113
**[4 °C 10 °C)**	−1.135	−1.172	−2.264	−1.107	−1.134	−1.107	−2.209	−1.122
	1.122	−1.136	−2.203⁎⁎	−1.124⁎⁎⁎	1.113	−1.110	−2.209	1.121
**[10 °C 21 °C)**	−1.12	−1.144	−2.244	−1.106	−1.118	−1.106	−2.206	−1.112
	−1.133⁎⁎⁎	1.218⁎⁎	2.285⁎⁎	1.191⁎⁎⁎	1.134	1.107	2.211	1.123
**[21 °C 27 °C)**	−1.11	−1.159	−2.292	−1.11	−1.124	−2.217	−2.213	−1.125
	1.133⁎⁎⁎	1.214⁎⁎	2.274⁎⁎⁎	1.160⁎⁎⁎	1.273⁎⁎⁎	2.287⁎⁎⁎	2.258⁎⁎⁎	1.135
**[27 °C 32 °C)**	−1.187	−1.183	−2.28	−1.113	−1.153	−2.23	−2.21	−1.124
	1.156⁎⁎⁎	1.126	2.212⁎⁎⁎	1.124⁎⁎⁎	1.287⁎⁎⁎	3.348⁎⁎⁎	2.201⁎⁎⁎	1.161⁎⁎
**[32 °C above)**	−1.196	−1.147	−1.19	−1.105	−1.152	−2.238	−2.209	−1.131
	1.189⁎⁎⁎	−4.453	1.102⁎⁎	1.108⁎⁎⁎	1.152⁎⁎⁎	3.324⁎⁎⁎	2.264⁎⁎⁎	1.151
**No of Observation**	2715	2169	2192	1182	12,338	4134	4269	2313
**Adj-*R2***	0.412	0.418	0.411	0.405	0.421	0.423	0.410	0.392
**County FE**	Yes	Yes	Yes	Yes	Yes	Yes	Yes	Yes
**FE Data, by province, annual**	Yes	Yes	Yes	Yes	Yes	Yes	Yes	Yes
**Climate Regulations**	Yes	Yes	Yes	Yes	Yes	Yes	Yes	Yes
**Label for Energy Effectiveness**	Yes	Yes	Yes	Yes	Yes	Yes	Yes	Yes
**Population Regulation**	Yes	Yes	Yes	Yes	Yes	Yes	Yes	Yes
**Home Automation Systems**	Yes	Yes	Yes	Yes	Yes	Yes	Yes	Yes

The impact of temperature is greatly intensified in the southern region of China. The reported surge in power usage for a mean temperature above 32 °C (28.9 %) is not supported by data. The weather conditions in southern China may be responsible for this amplification. In contrast, natural ventilation is less effective in controlling humidity levels in households in that region [35], necessitating air conditioning systems and other cooling devices throughout the summer to avoid heat stress and overheating. Conversely, due to public heating, the power consumption of northern residents is insensitive to colder days, but they appear to be particularly vulnerable to heat stress. Moderate heat, such as an additional day in the 16–21 °C range, increases power usage since northern individuals are less resilient to heat stress. Since hot days are rare in northern China, it isn't easy to reliably evaluate the effects at higher temperatures. In addition, there is a marked disparity in the effect of temperature on urban versus rural environments. Estimates for urban homes in prefectural or higher-level cities are shown in column (3), while estimates for suburban areas outside major cities are listed in column (4). Compared to rural regions, urban regions encounter a threefold growth in the effect of severe climate on energy use because of the more significant occurrence of air conditioning use. Our investigation indicates that almost all urban households in our sample have air conditioning, averaging 0.782 units, whereas about a third of rural homes have air conditioning, averaging 0.265 units.

Furthermore, our significant findings are supported by the fact that urban families are immune to cold stress because public heating is only available in metropolitan areas. The urban-rural divide indicates the distributive impact of climate change, which disproportionately affects rural residents with limited access to public heating and fewer resources to handle severe weather. To better understand the distributional implications of extreme temperatures, we classify families into high- and low-income categories in columns (5) and (6). High-income families require more energy to combat hot weather. Unlike wealthy families, low-income families are unlikely to be severely impacted by extreme heat despite both groups facing the same problem. Finally, we examine whether the composition of homes affects the correlation between high temperatures and power usage. The results for households with children under 12 or adults over 60 are presented in column 7. The baseline gradient in Table 2 is quite similar to the response to severe temperatures. It is improbable that individuals less able to tolerate temperature variations will require less energy to sustain a comfortable indoor temperature. As illustrated in the eighth column, abodes are bereft of elderly individuals or juveniles with lower sensitivity towards intense temperatures. This discovery could be attributed to most of them being in the workforce and spending fewer hours at home.

### 3.3 Effect of extreme heat on electricity consumption

The temperature regulation of a building through air conditioning reduces heat stress, as per the studies [36,37]. The effects of heightened temperatures on electricity consumption were investigated, focusing on the impact of air conditioning during extreme and prolonged heat waves. The purchase of air conditioners may be affected by high temperatures for two reasons despite being temporary. Firstly, families may be tempted to invest in air conditioning due to the fixed cost and their desire for increased interior comfort. Secondly, people's perceptions of future temperatures may change after experiencing excessive heat [38,39]. Fearful families may opt to purchase air conditioners, possibly more than one, to protect themselves from future heat waves. Our evaluation used the wealth of information available on air conditioners in CRECS, which includes data on the prevalence of AC use in homes, the number of ACs being used, the power consumption of each AC, its efficiency, and the typical usage pattern of the family as in Eq. (2).

(2) $$AC_{ijt}=\sum_{m}\;\beta^{m}Temp_{jt}^{m}+\delta^{\prime }Z_{it}+\varphi^{\prime }S_{ijt}+\gamma^{\prime }F_{jpt}+\varepsilon_{ijt}$$

We embark on a study of AC possession in the outskirts, where AC_ijt_ is denoted as one if there exists at least a single AC in the household and 0 otherwise. Employing a linear probability model that can estimate many fixed effects, we resolve Eq. (3). Then, we approach AC ownership from a concentrated perspective, setting AC_ijt_ as the total number of ACs in the home. In contrast to many other countries, China's use of individual ACs for each bedroom is highlighted by Refs. [40,41] as a distinctive feature. According to CRECS, affluent homes can install up to five ACs. As the dependent variable is numeric, we use a form of the ordered logit model popularized by Refs. [[42], [43], [44]]. The defining characteristics on the right-hand side of the equation are not defined by Equation (1). The data is exhibited in Table 4. When the daily mean heat reaches 27 °C or more, temperature stress substantially increases the probability of possessing ACs at the extended and intense limitations.

**Table 4:** Mechanism outcomes.

	1	2	3	4	5	6	7	8
	InstalledAC? (Yes=1)	NumberofACs	ACaccumulative power	AC using Frequency	ACEnergy Efficiencyscore	Installed WH? Yes=1	WH using Frequency	WH Energy Efficiency score
**(below −11°)**	−1.138⁎⁎⁎	−2.392⁎⁎⁎	−1.211⁎	−5.420⁎⁎⁎	1.177	1.128	−1.219⁎⁎⁎	1.166⁎⁎
**[−11 °C -6 °C)**	1.121	−2.219⁎⁎	−1.373⁎	−1.592⁎⁎	−1.158	1.125⁎⁎	−1.277	−1.132
**[−6 °C -2 °C)**	−1.109	−1.801⁎⁎	−1.254	−2.398⁎⁎⁎	1.188	−1.135⁎⁎⁎	−1.243	1.102
	−1.109	−1.287	−1.174	−1.264	−1.181	−1.108	−1.281	−1.118
**[−2 °C 5 °C)**	1.102	−1.210	−1.115⁎⁎	−1.268	−1.136	1.137⁎⁎	−1.175⁎⁎⁎	1.102
	−1.107	−1.134	−1.198	1.166	−1.131	−1.114	−1.194	−1.113
**[5 °C 11 °C)**	−1.10	1.121	1.115	1.155	1.14	−1.104	−1.141	1.118⁎⁎
	−1.16	−1.162	−1.149	−1.105	−1.138	−1.108	−1.215	−1.108
**[11 °C 22 °C)**	1.103	1.118	−1.159	−1.107	−1.292⁎⁎⁎	−1.113	−1.265⁎⁎	−1.140⁎⁎⁎
	−1.109	−1.226	−1.202	−1.107	−1.129	−1.113	−1.17	−1.114
**[22 °C 28 °C)**	1.119	1.233⁎⁎	−1.229	1.191	−1.276⁎⁎⁎	1.129	−1.160	1.145
	−1.112	−1.266	−1.189	−1.127	−1.148	−1.124	−1.156	−1.13
**[28 °C 33 °C)**	1.136⁎⁎	1.885⁎⁎⁎	1.214⁎⁎⁎	1.393⁎⁎	−1.122⁎⁎⁎	1.116	−1.141	1.154⁎
	−1.115	−1.29	−1.199	−1.241	−1.158	−1.128	−1.206	−1.132
**[33 °C above)**	1.144⁎⁎	1.990⁎⁎⁎	1.247⁎	2.144⁎⁎	−1.343⁎⁎	−1.217	−1.212	1.108
	−1.117	−1.123	−1.108	−1.247	−1.408	−1.137	−1.277	−1.149
**No of Observation**	6426	6376	7764	2324	5151	6355	3516	5886
**Adj-*R2* [Pseudo *R2*]**	0.435	0.254	0.341	0.271	0.240	0.162	0.378	0.762
**Country FE**	Yes	Yes	Yes	Yes	Yes	Yes	Yes	Yes
**Yearly FE Data by province**	Yes	Yes	Yes	Yes	Yes	Yes	Yes	Yes
**Climate Regulations**	Yes	Yes	Yes	Yes	Yes	Yes	Yes	Yes
**Label for Energy Effectiveness**	Yes	Yes	Yes	Yes	Yes	Yes	Yes	Yes
**Population Regulations**	Yes	Yes	Yes	Yes	Yes	Yes	Yes	Yes
**Domestic Regulations**	Yes	Yes	Yes	Yes	Yes	Yes	Yes	Yes

These findings indicate that families will only invest in ACs if the risk of heat stress is significant. However, the likelihood of obtaining an AC and, more importantly, having several ACs decreases significantly due to exposure to cold stress. Table 1 illustrates that most cold days occur in northern China, where summers are considerably colder, so ACs are less needed. The third column examines whether increased demand for ACs because of warmer weather would result in purchasing more powerful units. Since some homes have more than one AC, we include all the ACs in the household's overall cooling capacity. Counties with frequent hot days have a higher percentage of families installing powerful ACs. The duration spent cooling indoor spaces during the summer has replaced the AC's power in column (4). Adopting ACs is a necessary but insufficient condition for temperature to affect domestic power usage. Thus, this activity is critical. The high cost of operating an AC has led some households, particularly those with elderly residents, to restrict its usage. Nevertheless, we still find that prolonged exposure to high temperatures necessitates using ACs.

Inquiring about the energy efficiency of air conditioners, we pondered whether people would pay more for eco-friendly models. It is improbable that households, faced with increasingly sporadic heatwaves, would be more inclined to consider purchasing energy-wasting air conditioning units, which may indicate that they have not made any strides in the battle against climate change. As people persist in prioritizing energy-saving home appliances, they are procuring more environmentally friendly air conditioners. The idea is that air conditioners are not the most energy-efficient option as they are typically replaced more often than laptops, refrigerators, and televisions. We conducted a study on the energy efficiency of PCs and found no significant impact on temperature bins. We do not, however, have enough data to discount this theory entirely. We also considered whether people installed air conditioners before climate change became a significant issue and found that even those late to embrace them prioritize interior comfort over energy costs. Showers are an alternative to air conditioning, but installing water heaters is not a sensible response to extreme temperatures. Our findings in Table 5 were not affected by other household appliances, except for computer ownership, which increased due to people moving their leisure time indoors during extreme temperatures.

**Table 5:** Results of household appliance ownership and energy efficiency.

	−1	−2	−3	−4	−5	−6	−7	−8	−9
	Have a TV? (Yes = 1)	No. of TVs	TV using frequency	Have a Refrigerator? (Yes = 1)	Refrigerator energy efficiency score	Have a WM? (Yes = 1)	WM energy efficiency score	Have a PC? (Yes = 1)	PC using frequency
**(below −11°)**	1.113⁎⁎	1.167	1.273⁎⁎⁎	−1.108	−1.195⁎	1.112	1.148⁎	1.108	1.277
	−1.107	−1.276	−1.152	−1.11	−1.15	−1.108	−1.129	−1.115	−1.489
**[−11 °C -6 °C)**	−1.107	−1.152	−1.241⁎⁎⁎	1.120⁎⁎	−1.120	1.101	−1.129	−1.109	−1.436
	−1.108	−1.207	−1.119	−1.11	−1.134	−1.106	−1.121	−1.107	−1.308
**[−6 °C -2 °C)**	1.109	1.204	1.188⁎⁎⁎	1.113	1.162⁎⁎	−1.102	1.124	1.107	−1.150
	−1.106	−1.202	−1.121	−1.113	−1.13	−1.104	−1.119	−1.104	−1.329
**[−2 °C 5 °C)**	1	1.208	−1.129⁎	−1.101	−1.142	1.104	−1.117	1.105	−1.120
	−1.104	−1.269	−1.116	−1.106	−1.126	−1.104	−1.115	−1.107	−1.186
**[5 °C 11 °C)**	1.107⁎⁎	1.218	1.159⁎⁎⁎	−1.110⁎⁎	−1.125	1.104	1.113	1.107⁎⁎	1.278⁎
	−1.103	−1.181	−1.107	−1.104	−1.116	−1.103	−1.111	−1.104	−1.2
**[11 °C 22 °C)**	−1.100	−1.193⁎⁎⁎	1.119	−1.111⁎	1.192⁎⁎	−1.101	1.107	−1.102	−1.179
	−1.105	−1.189	−1.118	−1.106	−1.136	−1.105	−1.116	−1.106	−1.232
**[22 °C 28 °C)**	1.101	1.123	−1.155⁎	−1.104	−1.106	1.105	−1.108	1.124⁎⁎⁎	1.26
	−1.105	−1.014	−1.131	−1.112	−1.126	−1.105	−1.125	−1.109	−1.103
**[28 °C 33 °C)**	1.101	−1.027	−1.152	−1.104	1.183⁎⁎	1.105	1.133	1.127⁎⁎⁎	1.284
	−1.105	−1.219	−1.132	−1.113	−1.136	1.05	−1.13	−1.109	−1.201
**[33 °C above)**	−1.102	−1.189	1.126	−1.116	1.139	−1.132	1.112	1.128⁎⁎⁎	1.583
	−1.106	−0.206	−1.149	−1.116	−1.171	−1.139	−1.142	−1.108	−1.362
**No of Observation**	7674	7674	7033	7674	7674	6491	4227	6255	2505
**Adj-*R2* [Pseudo *R2*]**	0.129	0.164	0.42	0.75	0.09	0.18	0.56	0.42	0.29
**County FE**	Yes	Yes	Yes	Yes	Yes	Yes	Yes	Yes	Yes
**Yearly FE Data by province**	Yes	Yes	Yes	Yes	Yes	Yes	Yes	Yes	Yes
**Climate Regulations**	Yes	Yes	Yes	Yes	Yes	Yes	Yes	Yes	Yes
**Label for Energy Effectiveness**	Yes	Yes	Yes	Yes	Yes	Yes	Yes	Yes	Yes
**Population Regulations**	Yes	Yes	Yes	Yes	Yes	Yes	Yes	Yes	Yes
**Domestic Regulations**	Yes	Yes	Yes	Yes	Yes	Yes	Yes		

### 3.4 Electricity use in the future, simulated

Table 6 summarizes the alterations expected for particular heat categories by each of the eight GCMs, considering various emission routes and time horizons. Appendix I lists anticipated changes for each of the ten temperature categories. It is foreseen that China will experience fewer cold days and more hot days due to the inevitable warming. The SSP126 trajectory foretells a negligible escalation of 0.21–0.42 days in the count of days with temperatures over 32 °C in the foreseeable future. Still, there are no significant variations expected in the long run. The probability of the SSP585 framework leading to a rise of 0.32–1.02 days with temperatures exceeding 32 °C in the medium term and 0.70–2.42 days, in the long run, is low, despite its lack of focus on reducing GHG emissions. These changes are significant compared to the average number of days with excessive heat in the counties evaluated by CRECS from 2018 to 2021, which was 1.524.

**Table 6:** Future domestic power demand forecasting.

Pathways	SSP125									
	Medium-term (2042–2061)					Long-term (2062–2081)				
GCMs	Δbin2	ΔBin7	ΔBin8	ΔBin9	ΔE	Δbin2	ΔBin7	ΔBin8	ΔBin9	ΔE
BCC-CSM2-MR	−1.21	−1.29	2.25	1.27	**4.39**	−1.17	−1.28	2.23	1.23	**7.67**
CNRM-CM6–1	−1.22	−1.20	2.38	1.23	11.47	−1.22	−1.11	2.327	1.26	16.51
CNRM-ESM2–1	−2.36	−2.33	2.21	1.19	14.39	−1.10	−3.38	3.34	1.27	14.47
CanESM5	−1.15	1.13	2.32	2.32	**15.43**	−1.17	1.12	2.36	2.39	**13.43**
IPSL-CM6A-LR	−1.25	1.18	2.35	2.34	15.52	−5.47	1.1	2.34	2.16	14.4
MIROC-ES2L	−2.18	1.18	2.22	1.13	21.24	−1.27	2.1	2.22	1.14	12.36
MIROC6	−1.16	−1.19	2.13	2.11	2.34	−1.16	−1.18	2.3	1.14	12.36
MRI-ESM2–0	−1.14	−2.35	2.15	1.18	13.4	−1.19	−1.15	2.15	1.18	11.14
Mean prediction	−1.034	−1.009	2.101	2.196	12.31	−2.044	−1.173	2.289	1.121	12.28

**Pathways**	**SSP586**									
	**Medium-term (2042**–**2061)**					**Long-term (2062**–**2081)**				
**GCMs**	**Δbin2**	**ΔBin7**	**ΔBin8**	**ΔBin9**	**ΔE**	**Δbin2**	**ΔBin7**	**ΔBin8**	**ΔBin9**	**ΔE**
BCC-CSM2-MR	−1.13	−1.27	2.64	1.5	16.54	−1.28	−1.22	4.43	2.39	24.31
CNRM-CM6–1	−1.13	−1.13	2.73	1.36	14.32	−1.21	−1.27	1.15	2.16	31.11
CNRM-ESM2–1	−1.29	−1.10	2.59	1.34	12.29	−1.27	−1.25	1.37	2.17	21.18
CanESM5	−1.21	−1.16	1.52	3.21	**31.19**	−1.38	−1.13	2.27	2.12	**46.6**
IPSL-CM6A-LR	−1.17	1.17	1.13	2.37	21.11	−1.29	−1.17	1.17	2.18	33.21
MIROC-ES2L	−1.16	1.12	2.33	1.22	12.27	−1.28	1.15	1.19	2.17	24.45
MIROC6	−1.27	−1.20	2.32	1.23	**12.44**	−1.24	−1.17	1.1	2.1	**26.62**
MRI-ESM2–0	−1.27	−1.23	3.4	1.38	12.2	−1.18	−1.13	1.38	2.21	24.47
Mean prediction	−1.141	−1.225	2.445	1.239	18.424	−1.204	−1.199	1.345	2.238	32.28

By joining our baseline approximations in column (6) of Table 2 with the expected shifts in daily temperatures, we have created a model to predict the changes in China's home power consumption. To rate the projected increase in household power usage as credible based on the most sustainable scenario. Conversely, should we persist with the existing situation, we can anticipate household power usage growth of 13.94–30.09 % and 23.82–47.70 % in the average and longer term. These significant differences in anticipated home power consumption highlight the benefits of reducing GHG emissions to decrease domestic electricity usage, reliable with the most supportable approach.

## 4 Robustness checks

To ensure the dependability of our outcomes, we conduct numerous tests. Initially, we follow [45] lead by selecting a temperature bin reference range of 16–21 °C, which is higher than the literature's commonly accepted 10–16 °C range. Families residing in the north typically favor the temperature range of 10–16 °C, whereas those in the South are more comfortable with temperatures between 16 and 21 °C. Alternatively [46,47], proposes a benchmark range of 15.6–18.3 °C for mild climates, which we find our results to be robust against. In the second step, we decrease the temperature bin sizes from 6 to 3 °C to provide greater flexibility in evaluating temperature impacts.

Thirdly, we create temperature bins to separate the effects of maximum daily temperature. Our estimates, as demonstrated in Table 7, are reliable with our baseline outcomes based on mean temperatures, albeit lower. The reduced estimates can be justified because the daily maximum temperature may not fully capture the heat stress exposure throughout the day, as unveiled by Refs. [48,49]. In the fourth step, we consider various standard error clustering assumptions.

**Table 7:** Changes in the daily distribution of mean temperatures 2041–2060 SSP126.

GCMs	(below −12 °C)	[-12 °C −7 °C)	[-7 °C −1 °C)	[-1 °C 4 °C)	[4 °C 10 °C)	[10 °C 16 °C)	[16 °C 21 °C)	[21 °C 27 °C)	[27 °C 32 °C)	[32 °C above)
BCC-CSM2-MR	−0.11	0.01	−0.18	−0.25	0.05	−0.23	−0.31	−0.49	1.15	0.37
CANESM5	−0.25	−0.16	−0.47	−0.59	−0.27	−0.04	−0.38	0.03	1.72	0.42
CNRM-CM6-1	−0.12	−0.06	−0.32	−0.40	0.03	−0.18	−0.46	−0.20	1.38	0.33
CNRM-ESM2-1	−0.16	−0.06	−0.29	−0.26	0.08	−0.32	−0.45	−0.13	1.31	0.29
IPSL-CM6A-LR	−0.15	−0.02	−0.22	−0.35	−0.13	−0.30	−0.51	0.08	1.35	0.24
MIROC6	−0.06	−0.02	−0.21	−0.31	−0.04	−0.13	−0.38	−0.09	1.03	0.21
MIROC-ES2L	−0.18	−0.08	−0.33	−0.47	−0.04	−0.03	−0.29	0.08	1.12	0.23
MRI-ESM2-0	−0.04	0.00	−0.15	−0.32	−0.17	−0.32	−0.50	−0.15	1.35	0.28

Column (2) initiates "several city-level variables, including PM_2.5_ concentrations, three-year moving average population growth", middle school student-teacher ratios, and physicians per 10,000 residents. Except for "PM_2.5_ concentrations", all data is sourced from "China City Statistic Yearbooks". We generated " PM_2.5_ concentrations using satellite AOD retrievals and the method" [50,51] due to the absence of a nationwide PM_2.5_ monitoring network until early 2014 [52,53]. have furnished compelling evidence to demonstrate the effect of city-level characteristics on the sorting patterns of Chinese households. Fortunately, our findings persist even after incorporating them into our favored setup. We utilize "regional temperature disturbances" in temperature categories to pinpoint the root cause of temperature irregularities. Our technique for identifying such disturbances relies on the observation that temperatures frequently experience temporary fluctuations before reverting to their long-term average. The identification is founded upon the concept that long-term average temperatures might be impacted by energy consumption, whereas abrupt changes are exogenous due to their unexpected nature. The following attributes typify "local shocks" as in Eq. (3):

(3) $${\text{local}\;\text{shock}}_{jt}^{m}={\text{Temp}}_{jt}^{m}-\frac{\sum_{Y=1980}^{2014}\;Temp_{jY}^{m}}{35},t\notin Y$$

in the county of J, during the years spanning from 1980 to 2014, the local shock_jt_ records the anomalies of the maximum heat bin from its norm. Our methodology, inspired by Refs. [[54], [55], [56]], involves disregarding the weather information of year t when calculating the long-term mean days in the bin for computing the short-term deviation. The results expounded in Appendix F corroborate our initial hypotheses. Our research reveals that families tend to increase their power consumption in response to heat shocks, but the reaction to cold shocks is relatively weaker, which aligns with the fact that homes in northern China rely on public heating. We utilize [[57], [58], [59]] bounding method to determine the robustness of our chosen specifications to missing data. The method proposed by Ref. [60] employs shifts in the "coefficient of interest and the coefficient of determination (R2)" in the manipulated and unmanipulated models to infer the impact of missing data [60]. puts forth a practical way of producing boundaries on the "coefficient of interest". The coefficient of interest is deemed free of any omitted variable bias if its confidence interval does not include zero. The OLS estimate limits are presented in Appendix G for each temperature category. The outcomes from the complete specification are illustrated in Table 2, column (1), and the bounds for the calculated coefficient are presented in column (2). The temperature bin [7 °C–1 °C] is the only category that has a border that includes zero. These test results raise doubts that unobservable factors drive the primary findings.

## 5 Conclusion and policy implications

Through a novel survey utilizing verified electricity consumption data, we have determined the impact of extreme temperatures on household power usage in China, the world's largest electrical market, which obtains over 70 % of its energy from fossil fuels. We have divided the yearly distribution of daily mean temperatures into ten separate bins, each of which can be used to estimate temperature. Our results show an asymmetrical correlation between temperature and energy use, as homeowners use more power during hot days when they turn up their air conditioning. Families in northern China, where public heating is provided, are mainly responsible for this outcome, as they are less responsive to freezing temperatures. Moreover, impoverished families, particularly those dwelling in rural areas, are unlikely to take action when faced with intense heat. Our analysis proves that the prevalent and substantial use of air conditioners is the key driving force behind our discoveries since individuals frequently upgrade to more effective air conditioners during hot weather. This implies that households have changed their purchasing behaviors to limit the effects of climate change. Our research predicts that, under a routing scenario (SSP585) where no efforts are made to reduce GHG emissions, Chinese families would use an additional 47.7 % of power to combat heat stress by the end of the 2080s. However, in a sustainable scenario (SSP126), the increase in power usage would be just 9.77 %.

The implications of our research bear substantial policy relevance, particularly in the imperative need to curtail greenhouse gas (GHG) emissions to alleviate the impact of heat stress on household energy consumption. In alignment with China's commitment under the Paris Climate Accord, the nation's transition towards eco-friendly energy sources and reduced reliance on coal is an established commitment. Offering financial assistance to economically challenged households could alleviate climate change's effects, given that financially vulnerable families are more susceptible to the consequences of rising temperatures.

As air conditioners play an increasingly crucial role, particularly for low-income families, it is suggested that reducing the cost of these units could enhance the quality of life amidst climate change. Government support and minimizing operational expenses are deemed essential to address the substantial power consumption of air conditioning. Optimizing the efficiency of air conditioning units, with a focus on promoting the use of advanced, energy-efficient models, presents a viable solution. Decision-makers are encouraged to expedite the phasing out of outdated, less efficient air conditioning units, as endorsed by the International Energy Agency (IEA).

To ensure long-term reductions in household power consumption without compromising comfort, we recommend elevating energy efficiency standards for cooling equipment and optimizing building ventilation architecture. Our findings indicate a willingness among households, especially in southern China, to upgrade to more efficient air conditioners. In regions where public heating is under consideration, the Chinese government should prioritize renewable energy sources, considering local climatic conditions to prevent energy waste. This shift aligns with the government's commitment to reducing greenhouse gas emissions and addresses the environmental and health risks associated with heavy coal reliance in northern China.

We propose allocating a larger share to renewable energy sources in electricity, given the anticipated rise in energy consumption, particularly in developing nations. While fossil fuels may still be necessary for certain energy requirements, increased pollution, and carbon emissions are likely. We recommend further investment in renewable sources like wind and hydropower to address peak electricity demand during hot summer days, which strains the power grid. Despite the prevailing assumption, these sources are often better equipped to handle weather irregularities than fossil fuel-based alternatives. Furthermore, the potential of power storage technology, exemplified by Tesla's large-scale lithium-ion battery, demonstrates the feasibility of storing renewable energy in advance and releasing it during peak demand.

However, our study has limitations, including using a cross-sectional dataset with recurring samples. We acknowledge constraints in capturing the diverse dynamics within households over time, hindering a comprehensive understanding of homes' transient response to extreme temperatures. While robustness tests have been employed, future research could benefit from exploring household panel data to enhance the nuanced understanding of energy consumption patterns and contribute to more effective climate change mitigation strategies.

## Ethics approval and consent to participate

Not applicable.

## Consent for publication

All of the authors consented to publish this manuscript.

## Funding

Academic funding project Ministry of education of the PRC in 2022 under Grant:A Study on Deep-Sea Politics and National Three-dimensional New Sea Power under China's Maritime Strategy (No. 22YJCZH152).

## Data availability

We collected relevant data from World Bank open data available at https://data.worldbank.org/. For any further query on data, corresponding author at email address suyongsam@sina.com may be approached.

## CRediT authorship contribution statement

**Yong Su:** Writing – review & editing, Writing – original draft, Visualization, Resources, Methodology, Investigation, Formal analysis, Data curation, Conceptualization. **Kaleem Ullah:** Writing – review & editing, Writing – original draft, Investigation, Funding acquisition, Data curation, Conceptualization.

## Declaration of competing interest

The authors declare that they have no known competing financial interests or personal relationships that could have appeared to influence the work reported in this paper.
